# 

**Published:** 1864-04

**Authors:** 


### CHICAGO MEDICAL JOURNAL.

**Terms, $2.00 per Annum.**

### CONTENTS OF THE APRIL NUMBER.

PAGE.

ORIGINAL COMMUNICATIONS.

Rambles in Military Hospitals —Prison Hospital, Rock Island, Ill. By R. M. Lackey, M. D., A. A. Surg. U. S. A........ 145
On the Treatment of Empyema by Chassagnac's Drainage Tube. By W. D. Slater, M D., of Chicago, Graduate of Royal College of Physicians of England, etc........ 149
Clinical Lectures on Diseases of the Eye. By E. L. Holmes, M. D., of Chicago, Lecturer on Diseases of the Eye and Ear in Rush Medical College, etc.—The Cornea—Injuries........ 152
Proceedings of Medical Societies........ 157

SELECTED.

Physiology and Pathology the True Foundation for Medical Practice. Extracts from a Lecture by John Hughes Bennett, M. D........ 160
The Consumptive Curers of New York. By an Invalid M. D........ 164
Mineral Acids in Dysentery. By Dr. James Henderson, Medical Officer to the Chinese Mission Ho-pital, Shanghai........ 168
On Gall Stones. By Dr. J. L. W. Thudichum........ 170
Efficacy of Sesquichloride of Iron for the Treatment of Ulcers about the Nails. By Dr. Billon, Surgeon 19th Regt........ 173
On the treatment of Eczema. By Dr. J. Wallace, Dalston, Carlisle........ 174
On the Treatment of Rheumatic Fever. By Willoughby F. Wade, M. D., M. R. C., Senior Physician to the Queen's Hospital, Birmingham... 175

EDITORIAL CORRESPONDENCE........ 180
BOOK NOTICES AND REVIEWS........ 186
Obituary........ 189

